# Desert Endophytic Fungi Differentially Modulate Reactive Oxygen Species and Antioxidant Responses to Heat Stress in Tomato

**DOI:** 10.3390/plants15152311

**Published:** 2026-07-28

**Authors:** Yessica San Miguel, Pedro E. Gundel, Luis Morales-Quintana, Patricio Ramos

**Affiliations:** 1 Plant-Microorganism Interaction Laboratory, Institute of Biological Sciences, University of Talca, Talca 3460787, Chile; yessica.sanmiguel@utalca.cl; 2 Doctorado en Ciencias Mención Biología Vegetal y Biotecnología, Universidad de Talca, Talca 3460787, Chile; 3 Instituto de Biología Funcional y Biotecnología (BIOLAB)-INBIOTEC-CONICET-CICBA, Facultad de Agronomía, Universidad Nacional del Centro de la Provincia de Buenos Aires (UNICEN), Tandil B7000GHG, Argentina; gundel@agro.uba.ar; 4 Centro de Ecología Integrativa, Instituto de Ciencias Biológicas, Universidad de Talca, Talca 3460787, Chile; 5 Multidisciplinary Agroindustry Research Laboratory, Instituto de Ciencias Biomédicas, Universidad Autónoma de Chile, Talca 3460787, Chile

**Keywords:** oxidative stress, ROS homeostasis, antioxidant enzymes, phenolic compounds, flavonoids, plant–microbe interactions, stress tolerance

## Abstract

Increasing temperature is a major stress factor associated with climate change, strongly limiting agricultural productivity, particularly in sensitive crops such as tomato (*Solanum lycopersicum* L.). High temperatures promote the accumulation of reactive oxygen species (ROS), leading to oxidative damage and disruption of key physiological processes. Here, we evaluated whether desert-derived endophytic fungi differentially modulate oxidative stress responses in tomato plants exposed to heat stress. Plants were inoculated with either *Talaromyces minioluteus* or *Serendipita indica* and grown under control (22/19 °C) or heat stress conditions (35/19 °C). Heat stress reduced growth and increased oxidative damage, whereas endophyte inoculation mitigated these effects. Inoculated plants showed higher shoot and root biomass, lower levels of malondialdehyde (MDA) and hydrogen peroxide (H_2_O_2_), and enhanced enzymatic and non-enzymatic antioxidant systems, including increased activities of superoxide dismutase (SOD), catalase (CAT), and ascorbate peroxidase (APX), as well as greater accumulation of phenolic compounds and flavonoids. However, the magnitude and direction of these responses depended on the endophyte species. Overall, endophytic fungi modulated ROS homeostasis through coordinated enzymatic and non-enzymatic antioxidant mechanisms. These findings indicate that desert endophytic fungi enhance tolerance to heat stress through endophyte-specific regulation of oxidative balance, highlighting their potential to improve crop resilience under climate change.

## 1. Introduction

Tomato (*Solanum lycopersicum* L.), a member of the Solanaceae family, is one of the most widely cultivated vegetables worldwide, valued for its nutritional and economic importance [1,2]. According to FAOSTAT [3], tomato is the second most important vegetable crop globally, after potato. Tomatoes are a rich source of minerals, vitamins A and C, and phytochemicals such as β-carotene, lycopene, and flavonoids [4], which contribute to their high consumption and market demand [5]. Tomato production is typically intensive, often conducted in relatively small fields or under greenhouse conditions, where crops are highly managed and depend on controlled environmental conditions to achieve optimal yields [6].

Despite this level of management, tomato production systems remain highly vulnerable to extreme climatic events. In the current context of global warming, heatwaves and water scarcity are increasingly affecting plant performance and productivity [7]. Heatwaves are projected to become more frequent and prolonged, particularly during summer [8], posing a significant challenge even in irrigated or protected systems, where short periods of high temperature can severely impair crop development and yield. These conditions are expected to reduce tomato productivity, with a projected decrease of approximately 6% by 2050 relative to the 1980–2009 period [9], and substantial yield losses reported under extreme heat stress conditions [10,11].

High temperatures represent a major abiotic stress affecting multiple stages of plant growth and development. Heat stress induces the rapid accumulation of reactive oxygen species (ROS), protein denaturation, alterations in enzymatic activity, and membrane instability [12,13]. Although ROS play important roles in signaling and stress perception, their overproduction leads to oxidative stress, causing damage to lipids, proteins, and DNA [7,14]. Under stress conditions, ROS and lipoxygenase activity promote lipid peroxidation, leading to the formation of malondialdehyde (MDA), a marker of oxidative damage whose levels reflect the extent of membrane deterioration and cellular injury [15]. To counteract these effects, plants deploy antioxidant systems, including non-enzymatic compounds such as flavonoids, ascorbic acid, and glutathione, as well as antioxidant enzymes such as superoxide dismutase (SOD), catalase (CAT), peroxidases (POD), and ascorbate peroxidase (APX), which together maintain cellular redox homeostasis [16]. In tomato, disruption of this redox balance under heat stress is associated with impaired germination, reduced vegetative growth, altered reproductive development (e.g., decreased flower number and pollen viability), and compromised fruit development, ultimately leading to yield losses [17].

To mitigate the adverse effects of abiotic stress on cultivated plants, several strategies have been proposed, including the use of tolerant cultivars, agronomic adjustments, and chemical treatments [7]. However, these approaches may be limited by economic cost and environmental impact [18]. An alternative approach involves exploiting beneficial plant-microorganism associations, particularly with endophytic fungi and bacteria adapted to extreme environments [18,19,20,21]. Some of these microorganisms can enhance plant tolerance to abiotic stress, including drought and high temperatures [22]. Although the mechanisms are not fully understood, endophytic fungi are thought to improve stress tolerance through multiple pathways, including the synthesis of osmolytes and secondary metabolites, modulation of phytohormone signaling, regulation of antioxidant systems and ROS levels, and induction of heat shock proteins (HSPs) [23,24]. According to these mechanisms, it has been shown that *Talaromyces minioluteus* isolated from quinoa crops from the Atacama Desert, Chile [25], and *Serendipita indica* isolated from desert plants (Thar Desert in India) [26] can improve the tolerance of plants to abiotic stress, including salinity and water deficit [27]. Similarly, endophytic fungi from extreme environments have been shown to enhance heat tolerance in different plant species, such as *Thermomyces* sp. in *Cucumis sativus* [28], and *Aspergillus japonicus* in soybean and sunflower, where improved antioxidant activity and accumulation of protective metabolites were observed [29].

Despite growing evidence of the beneficial effects of endophytic fungi under abiotic stress, the mechanistic basis of their role in modulating plant oxidative responses remains incompletely understood. Moreover, although some endophytic fungi exhibit broad host ranges and are considered generalists [30], the outcome of the symbiosis depends on the specific plant-fungus combination and is strongly modulated by environmental context [31]. Here, we investigated how the endophytic fungi *T. minioluteus* and *S. indica* influence oxidative stress responses in tomato under heat stress, with particular emphasis on antioxidant systems. We predicted that these endophytes may produce similar effects on plant performance but differ in the underlying biochemical and antioxidant mechanisms regulating oxidative balance under heat stress. These findings aim to inform the use of endophytic fungi as tools to enhance stress tolerance by highlighting the importance of matching fungal partners with specific host and environmental conditions.

## 2. Results

### 2.1. Effects of Heat Stress and Endophytic Inoculation on Plant Biomass and Allocation

**Figure 1:**
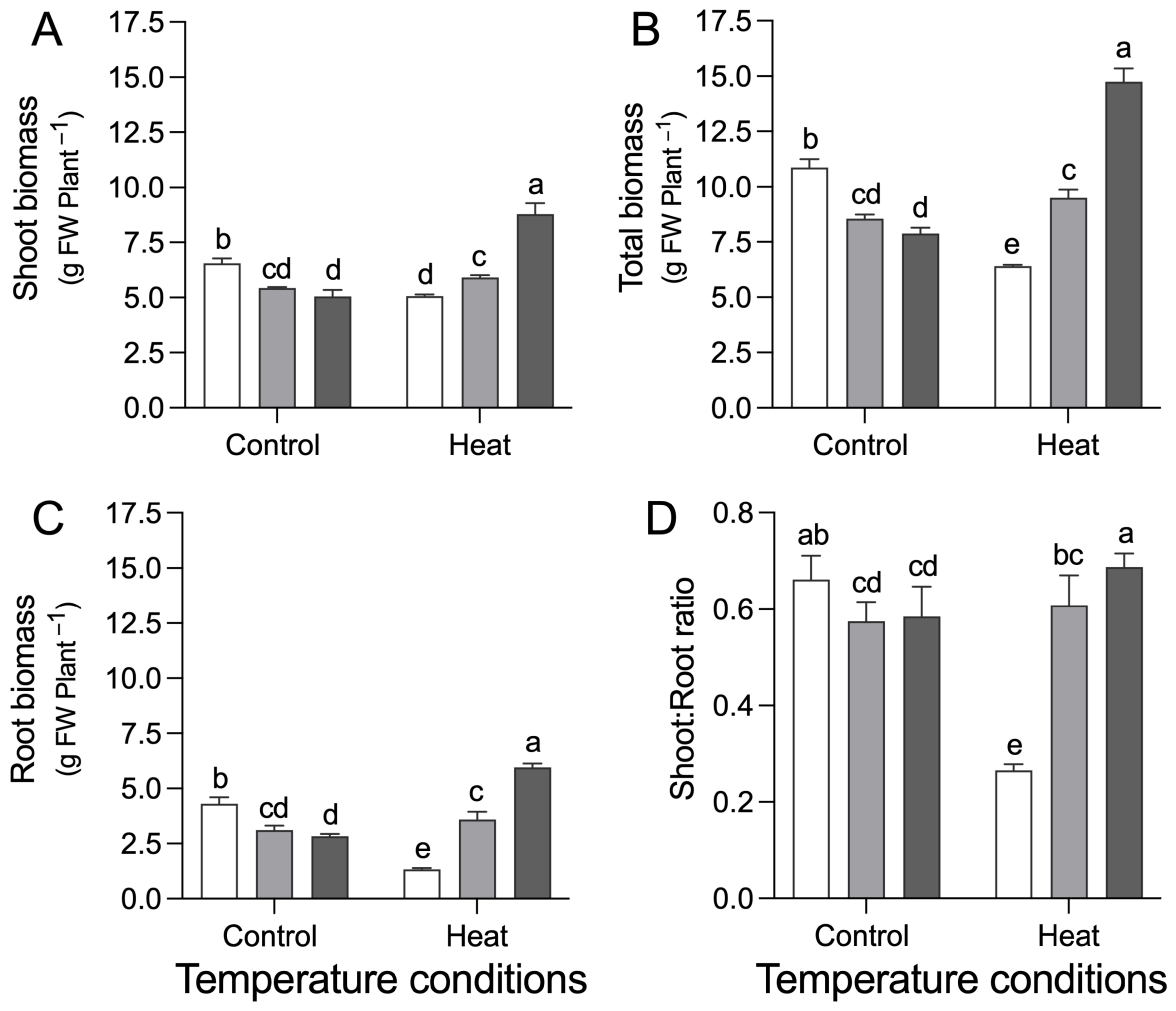
Shoot biomass (**A**), total biomass (**B**), root biomass (**C**), and shoot:root ratio (**D**) of *Solanum lycopersicum* plants either non-inoculated (E−, white bars) or inoculated (E+) with the root colonizing endophytic fungi *Talaromyces minioluteus* (E1+, grey bars) and *Serendipita indica* (E2+, black bars), grown under two temperature conditions (Control: 22 °C; heat stress: 35 °C). Different letters indicate significant differences among inoculation × temperature combinations (Tukey’s HSD, *p* ≤ 0.05).

The effects of heat stress on total, shoot, and root biomass, as well as the shoot:root ratio, depended on inoculation, as indicated by a significant inoculation × heat stress interaction (*p* < 0.001). Non-inoculated plants exposed to 35 °C showed significantly reduced total, shoot, and root biomass compared with control conditions (Figure 1A–C). In this case, the marked reduction in the shoot:root ratio indicates that shoots were more severely affected than roots (Figure 1D). This negative effect of heat stress on biomass was not observed in inoculated plants. Plants inoculated with *T. minioluteus* (E1+) maintained growth at 35 °C, whereas those inoculated with *S. indica* (E2+) showed greater growth at 35 °C than at 22 °C (Figure 1A–C). Inoculated plants maintained a stable shoot:root ratio, indicating that above- and belowground tissues were similarly affected (Figure 1D).

### 2.2. Effects of Heat Stress and Endophytic Inoculation on Oxidative Stress Markers

**Figure 2:**
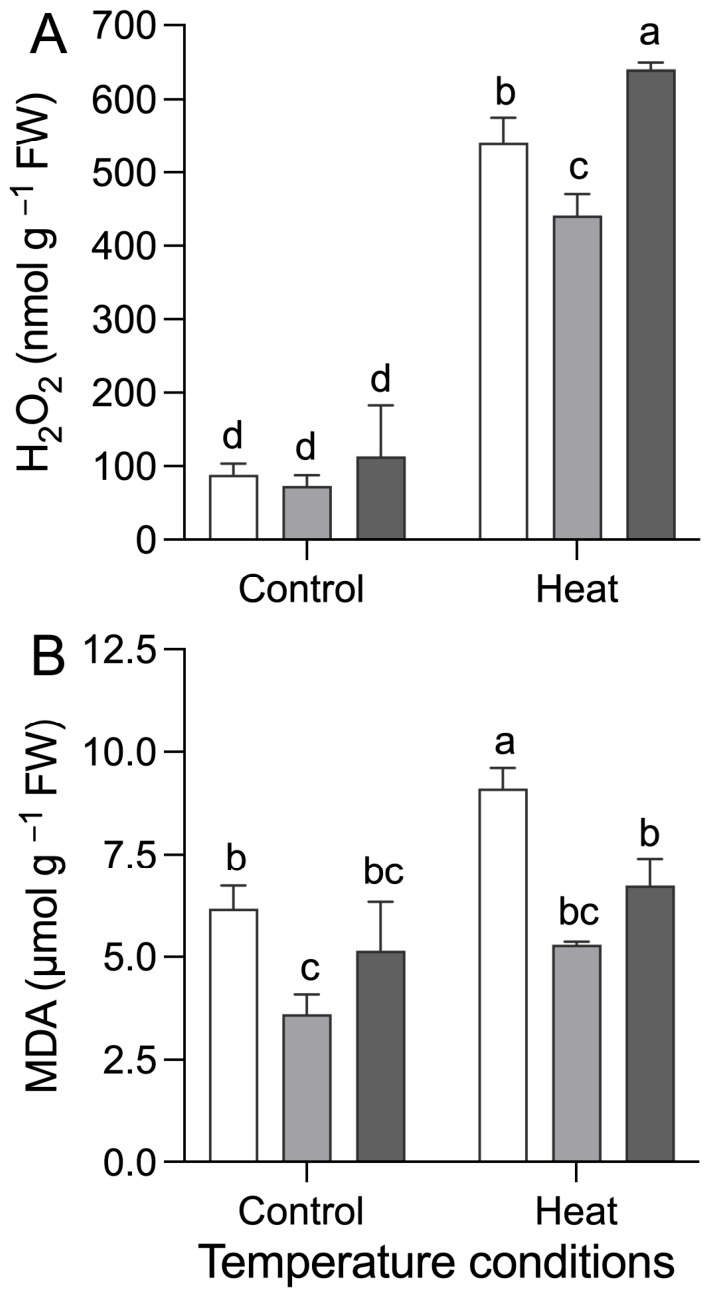
Hydrogen peroxide (H_2_O_2_) content (**A**) and malondialdehyde (MDA) content (**B**) in *Solanum lycopersicum* plants either non-inoculated (E−, white bars) or inoculated with the root-colonizing endophytic fungi *Talaromyces minioluteus* (E1+, grey bars) and *Serendipita indica* (E2+, black bars), grown under two temperature conditions (Control: 22 °C; heat stress: 35 °C). H_2_O_2_ was used as an indicator of reactive oxygen species accumulation, and MDA as an indicator of lipid peroxidation. Different letters indicate significant differences among inoculation × temperature combinations (Tukey’s HSD, *p* ≤ 0.05).

The effect of heat stress on H_2_O_2_ levels depended on fungal inoculation (*p* < 0.001). While H_2_O_2_ levels did not vary with inoculation under control conditions, they were significantly higher at 35 °C and differed among inoculation treatments (Figure 2A). Plants inoculated with *T. minioluteus* (E1+) showed lower H_2_O_2_ levels than non-inoculated plants, whereas those inoculated with *S. indica* (E2+) showed higher levels (Figure 2A). MDA content increased under heat stress in non-inoculated plants (*p* < 0.001), whereas inoculation with *T. minioluteus* (*p* < 0.001) significantly reduced MDA levels under control conditions; no significant reduction was observed with *S. indica*. At 35 °C, endophyte-inoculated plants showed significantly lower MDA content than non-inoculated plants, an effect observed with both endophytes (Figure 2B).

### 2.3. Effects of Heat Stress and Endophytic Inoculation on Antioxidant Enzyme Activity

**Figure 3:**
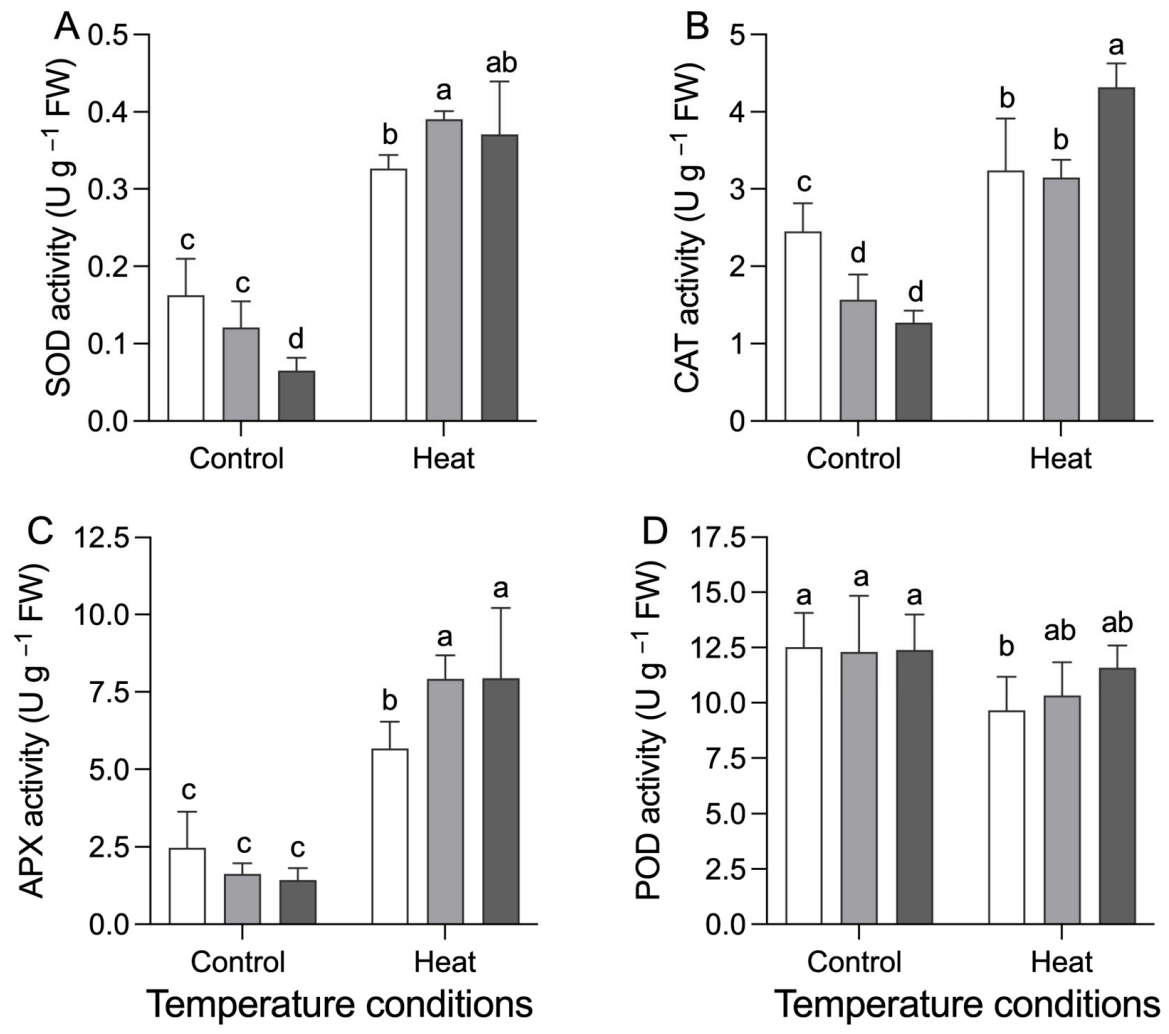
Antioxidant enzyme activities *in Solanum lycopersicum* plants either non-inoculated (E−, white bars) or inoculated with the root-colonizing endophytic fungi *Talaromyces minioluteus* (E1+, grey bars) and *Serendipita* indica (E2+, black bars), grown under two temperature conditions (Control: 22 °C; heat stress: 35 °C). Superoxide dismutase (SOD) activity (**A**), catalase (CAT) activity (**B**), and ascorbate peroxidase (APX) activity (**C**) and peroxidase (POD) activity (**D**), expressed as enzyme activity per gram of fresh weight (U g^−1^ FW). Different letters indicate significant differences among inoculation × temperature combinations (Tukey’s HSD, *p* ≤ 0.05).

The effect of heat stress on SOD, CAT, and APX activities depended on endophyte inoculation (*p* = 0.014, *p* = 0.005, and *p* < 0.001, respectively). Overall, enzyme activities increased under heat stress; however, both the magnitude and direction of this response depended on the inoculated endophyte (Figure 3A–C). Under control conditions, plants inoculated with *S. indica* showed the lowest SOD, CAT, and APX activities, while those inoculated with *T. minioluteus* exhibited intermediate activities, lower than those of non-inoculated plants. Under heat stress, endophyte inoculation generally increased SOD, CAT, and APX activities, except for *T. minioluteus*, which did not affect CAT activity relative to non-inoculated plants (Figure 3A,B). POD activity was significantly affected only by heat stress (*p* < 0.001) in plants without endophyte (white bars). Overall, POD activity was slightly lower under heat stress than under control conditions in inoculated plant; however, this reduction was not statistically significant (*p* = 0.211), suggesting that fungal endophytes are able to maintain POD activity under heat stress conditions (grey and black bars) (Figure 3D).

### 2.4. Effects of Heat Stress and Endophytic Inoculation on Non-Enzymatic Antioxidants

**Figure 4:**
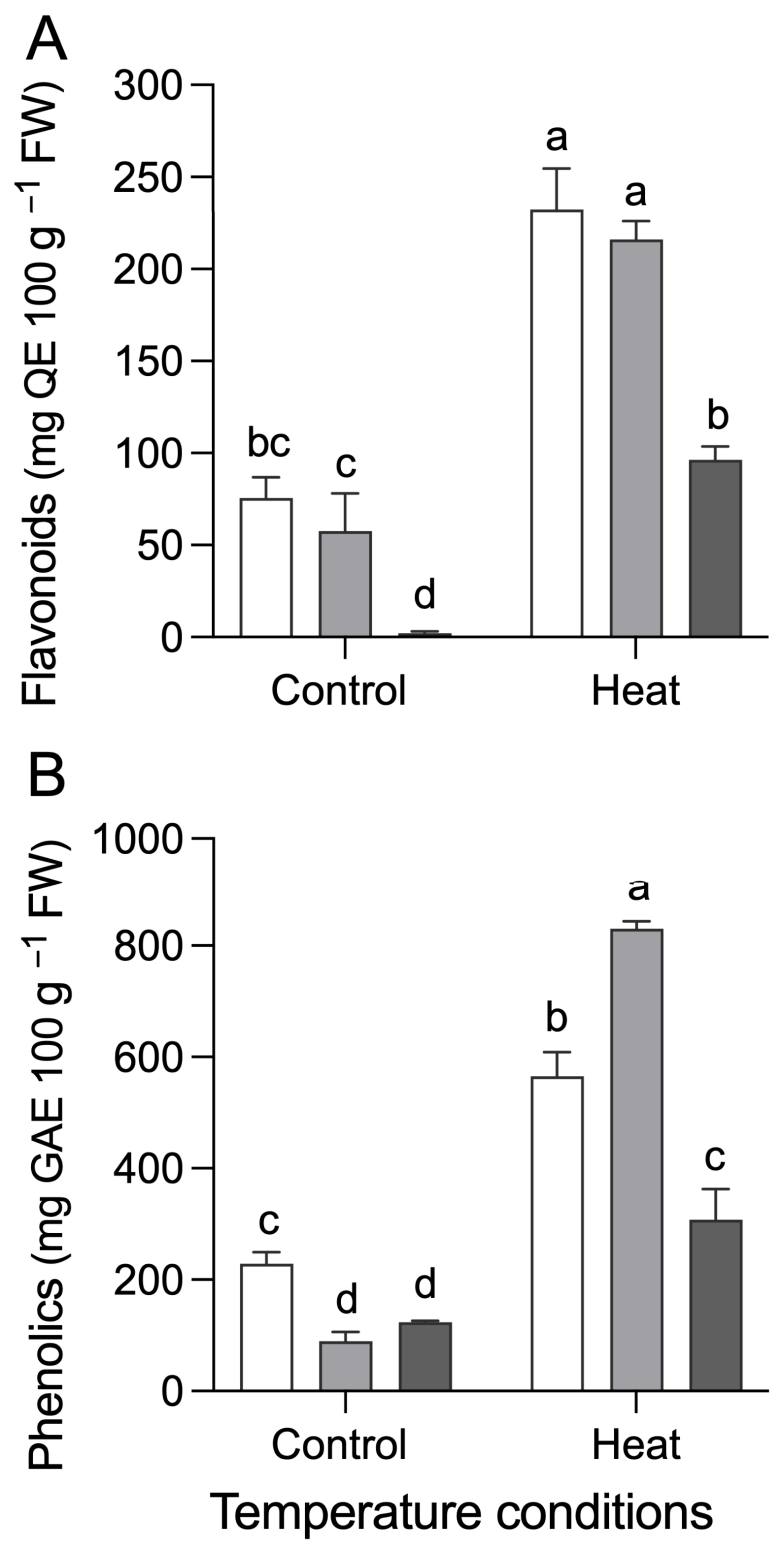
Non-enzymatic antioxidant compounds in *Solanum lycopersicum* plants either non-inoculated (E−, white bars) or inoculated with the root-colonizing endophytic fungi *Talaromyces minioluteus* (E1+, grey bars) and *Serendipita indica* (E2+, black bars), grown under two temperature conditions (Control: 22 °C; heat stress: 35 °C). Total flavonoid content (**A**) and total phenolic content (**B**). Different letters indicate significant differences among inoculation × temperature combinations (Tukey’s HSD, *p* ≤ 0.05).

The effect of heat stress on flavonoid and total phenol content depended on endophytic inoculation (*p* < 0.001). In general, both flavonoid and total phenol contents increased under heat stress (35 °C) compared to control conditions (22 °C). However, the magnitude of this increase varied among inoculation treatments. Under heat stress (35 °C), plants inoculated with *S. indica* (E2+) showed lower flavonoid content than non-inoculated (E−) plants and those inoculated with *T. minioluteus* (E1+) (Figure 4A). Similarly, plants inoculated with *T. minioluteus* (E1+) showed the highest total phenol content, differing from both non-inoculated (E−) and those inoculated with *S. indica* (E2+) (Figure 4B).

## 3. Discussion

Endophytic fungi from extreme environments represent a promising strategy to enhance crop tolerance to abiotic stress [18,19,20,21,22]. In this study, both *T. minioluteus* and *S. indica*, isolated from desert plants, successfully established symbiosis with tomato roots, as evidenced by the presence of intra- and intercellular hyphae (See Supplementary Material, Figure S1). Such colonization is a prerequisite for functional symbiosis and supports the concept of habitat-adapted associations, whereby microorganisms originating from extreme environments confer stress tolerance across a broad host range [19,30,32]. However, the effect of inoculation was strongly context-dependent; although endophytes reduced biomass under control conditions, they mitigated heat stress-induced reductions in plant performance, with the magnitude of this response differing between the two symbionts (Figure 1). This transition from negative to positive effects underscores the context dependency of symbiosis outcomes [31] and indicates that the successful application of extremophile fungi to enhance crop performance is case-specific and requires validation under field conditions [18].

Heat stress reduced biomass, particularly root biomass, in non-inoculated plants, confirming its detrimental effects on growth. However, inoculation with endophytic fungi mitigated these effects, with *S. indica* showing a stronger growth-promoting effect than *T. minioluteus*. The significant interaction between temperature and inoculation further indicates that plant responses to heat stress depend on symbiotic status, supporting the idea that endophytes contribute to stress acclimation through symbiosis-dependent mechanisms [19]. Differences between fungal species likely reflect variation in colonization efficiency, metabolite production, and signaling processes [33,34]. The stronger effect of heat stress on root biomass, together with the pronounced endophyte-mediated recovery in this compartment, suggests that tolerance involves the maintenance of root growth and function, encompassing resource acquisition as well as the physiological processes that sustain root performance under stress [35,36]. This effect may also involve endophyte-driven recruitment and assembly of the root-associated microbiome, which can further contribute to root growth and functioning, as well as to the modulation of plant defense mechanisms [37]. Importantly, the effects of endophytic inoculation may arise not only from direct influences on host physiology, but also from indirect mechanisms mediated through the modulation of plant–soil microbiome associations.

The endophyte-mediated mitigation of heat stress was closely associated with the regulation of oxidative damage. As expected, heat increased MDA levels across all inoculation treatments, indicating enhanced lipid peroxidation and membrane damage; however, endophyte inoculation reduced MDA accumulation, suggesting protection against oxidative stress. Interestingly, the control of MDA was higher in plants inoculated with *T. minioluteus* than in plants inoculated with *S. indica*. Despite this difference between endophytes, these results are consistent with previous studies showing that endophytic fungi reduce oxidative damage by modulating ROS levels and antioxidant responses [23,38,39]. However, the two fungi differed in their effects on H_2_O_2_ under heat stress; whereas *T. minioluteus* reduced the accumulation of H_2_O_2_, *S. indica* increased it. This contrast suggests that endophytes may regulate oxidative balance through distinct strategies, either by limiting ROS accumulation or by maintaining controlled ROS levels that may contribute to stress signaling. Notably, despite elevated H_2_O_2_ levels, *S. indica* exhibited enhanced plant growth under heat stress conditions, accompanied by reduced oxidative markers. This is evident in the decrease in malondialdehyde (MDA) levels, which is promoted by *S. indica* inoculation (Figure 2B).

Consistent with this interpretation, both enzymatic and non-enzymatic antioxidant systems were modulated by endophytic inoculation. The activities of SOD and CAT depended on the interaction between temperature and inoculation, indicating that endophytes enhance the capacity of plants to detoxify reactive oxygen species under stress. In contrast, POD activity responded primarily to heat stress and was not affected by endophyte inoculation, suggesting a more general response to temperature rather than inoculation-specific regulation. These enzymes play complementary roles in redox regulation, with SOD catalyzing the conversion of superoxide radicals into H_2_O_2_ and CAT subsequently decomposing H_2_O_2_ into water and oxygen [40,41]. In contrast, APX responded primarily to heat stress and was modulated by endophytes only under high temperatures, suggesting a more specific role in fine-tuning H_2_O_2_ detoxification [27]. In the case of POD, the reduction in activity caused by heat treatment was reversed by inoculation with each endophyte separately. In parallel, non-enzymatic antioxidants, specifically total phenolic compounds and the flavonoids, increased under heat stress and were differentially affected by fungal inoculation, further supporting the idea that redox homeostasis is regulated through coordinated antioxidant responses [42].

The decrease in POD activity, despite the activation of other antioxidant components, highlights the differential regulation of the antioxidant system under heat stress. In particular, the increased APX activity observed under specific temperature—inoculation combinations may have partially compensated for the lower POD activity, given the complementary contribution of these enzymes to H_2_O_2_ detoxification. Therefore, the reduced POD activity does not necessarily indicate a general impairment of antioxidant capacity but rather reflects the differential contribution of individual antioxidant components under heat stress. This interpretation is consistent with the capacity of endophytic fungi to modulate multiple antioxidant mechanisms, including enzymatic defenses and secondary metabolites, thereby contributing to the maintenance of redox homeostasis [23,27,42].

Importantly, the two endophytes differed in their modulation of antioxidant metabolism. *T. minioluteus* promoted higher accumulation of phenolic compounds, suggesting a stronger contribution of non-enzymatic antioxidant pathways, whereas *S. indica* showed a greater effect on biomass and enzymatic responses. These differences indicate that plant–endophyte associations are not functionally equivalent across fungal species but instead reflect distinct physiological strategies. Overall, our results highlight that endophytic fungi from extreme environments enhance heat stress tolerance in tomato by modulating oxidative balance, but through different mechanisms involving the integration of ROS regulation and antioxidant systems. Understanding these functional differences will be essential for the development of microbial-based strategies to improve crop resilience under climate change.

## 4. Materials and Methods

### 4.1. Biological Material and Growth Conditions

Tomato (*Solanum lycopersicum* L.) var. Micro-Tom seeds were surface-disinfected by immersion in 1% (*v*/*v*) sodium hypochlorite for 10 min, followed by three rinses with sterile distilled water to remove disinfectant residues [43], and germinated on moist filter paper at 28 °C for 2 days. Emerged seedlings were transplanted into 1 L pots containing a soil:peat mixture (1:1, *v*/*v*; supplemented with NPK (13-10-27%) fertilizer (0.90 g L^−1^). The substrate was autoclaved at 121 °C for 2 h [43]. Plants were maintained in a growth chamber under controlled conditions of 22/19 °C (day/night), a 16/8 h light/dark photoperiod, 70% relative humidity, and a photosynthetic photon flux density of approximately 700 μmol m^−2^ s^−1^.

### 4.2. Cultivation of Endophytic Fungi and Spore Collection

Two endophytic fungal strains isolated from plants growing in desert environments were used: *Talaromyces minioluteus* (E1+), isolated from the Atacama Desert (Chile) [25], and *Serendipita indica* (E2+), from the Thar Desert (India) [26]. The fungi were cultured on 0.1× potato dextrose agar (PDA) medium. Conidial suspensions were prepared and their concentration determined using a Neubauer chamber, then adjusted to 10^6^ conidia mL^−1^. Conidial viability was assessed following the methodology described by Greenfield et al. (2016) [44].

### 4.3. Experimental Design and Heat Stress Application

Plants approximately 9 weeks old (10–15 cm in height) were used. The experiment followed a completely randomized factorial design with two factors: (i) endophytic fungal inoculation and (ii) heat stress application. Endophytic inoculation included three levels: non-inoculated plants (control, E−), and plants inoculated with *T. minioluteus* (E1+) or *S. indica* (E2+). Each fungal strain was evaluated independently. The heat stress factor included two levels: control conditions (22/19 °C, day/night) and heat stress, in which plants were exposed to 35 °C for 4 h daily and then returned to optimal growing conditions. The temperature was gradually increased throughout the day, following a pre-established schedule, reaching 35 °C and maintaining this temperature for 4 h, before being progressively reduced to 19 °C at night. In detail, thermal oscillation increased by 0.5 °C per hour from 07:00 h to 12:00 h, after which the temperature remained constant at 22 °C for four hours. Subsequently, the temperature gradually decreased until it returned to 19 °C at 22:00 h. In the case of heat stress treatment, the temperature regimen was modified to a day-night cycle with a temperature oscillation of 35 °C/19 °C. The oscillation increased by 3 °C per hour from 07:00 h to 12:00 h, after which the temperature remained constant at 35 °C for four hours. Subsequently, the temperature gradually decreased until it returned to 19 °C at 22:00 h. Each treatment combination consisted of 10 replicates (one plant per pot).

Inoculation was performed by applying a suspension of conidia (1 × 10^6^ spores mL^−1^) resuspended in autoclaved distilled water directly to the rhizosphere. After inoculation, plants were maintained in a growth chamber under controlled conditions [22/19 °C (day/night); 16/8 h light/dark photoperiod; 70% relative humidity; photon flux density of 400 μmol m^−2^ s^−1^] for 15 days to allow symbiosis establishment, which was verified prior to stress treatments [See Supplementary Materials: Assessment of symbiosis establishment (Figure S1)].

Following the inoculation period, plants were allocated to either of the two climate chambers corresponding to each temperature treatment. Control plants were maintained under the same temperature regime (22/19 °C, day/night), while heat-stressed plants were exposed to 35 °C for 4 h daily for 31 days of treatment, as described above. Throughout the experiment, substrate moisture was maintained at field capacity, and pot positions within each chamber were randomly redistributed to minimize positional effects. At the conclusion of the experiment (31 days), leaf and root samples were collected at midday, coinciding with the temperature peak, immediately frozen in liquid nitrogen, and stored at −80 °C for all subsequent biochemical assays. The remaining shoot and root material was utilized to determine fresh weight. Each plant was characterized by total, shoot, and root biomass, as well as biomass allocation expressed as the shoot:root ratio.

### 4.4. Malondialdehyde (MDA) Content Determination

MDA content was determined using the thiobarbituric acid reactive substances (TBARS) assay following Barrera et al. (2020) [45]. Frozen plant tissue (100 mg), previously stored at −80 °C, was used. Tissue was homogenized in 2 mL of 0.1% TCA and centrifuged at 13,000 rpm for 15 min at 4 °C. The supernatant was transferred and mixed with 1.5 mL of 0.5% thiobarbituric acid (TBA) in 20% TCA. The mixture was incubated at 95 °C for 15 min and rapidly cooled on ice. Samples were centrifuged at 10,000 rpm for 10 min at 4 °C, and an aliquot (150 µL) of the supernatant was transferred to a microplate for absorbance measurement. Absorbance was recorded at 532 nm and non-specific absorbance at 600 nm using a microplate reader (Multiskan SkyHigh Microplate Spectrophotometer, Thermo Fisher Scientific, Waltham, MA, USA). MDA content was calculated using an extinction coefficient of 155 mM^−1^ cm^−1^.

### 4.5. Hydrogen Peroxide (H_2_O_2_) Content Determination

H_2_O_2_ content was performed following Chen et al. (2021) [46]. Fresh leaf tissue (500 mg) was collected from plants after 31 days of treatment, consistently at 13:00 h across all treatments, homogenized in 5 mL of ice-cold 0.1% (*w*/*v*) trichloroacetic acid (TCA), and the homogenate was centrifuged at 13,000 rpm for 15 min at 4 °C. An aliquot (0.5 mL) of the leaf supernatant was mixed with 0.5 mL of 100 mM potassium phosphate buffer (pH 7.0) and 1 mL of 1M potassium iodide (KI) and incubated in darkness for 1 h. Absorbance was measured at 390 nm using a spectrophotometer. H_2_O_2_ content was calculated using a standard curve generated with known H_2_O_2_ concentrations.

### 4.6. Antioxidant Enzymatic Activity Determination

Superoxide dismutase (SOD, EC 1.15.1.1) activity was estimated according to the method described by Erdogan et al. (2016) [47] by measuring the inhibition of nitroblue tetrazolium (NBT) reduction. An aliquot (200 µL) of enzymatic extract was added to 3 mL of 50 mM phosphate buffer (PBS, pH 7.8) containing 2 μM riboflavin, 13 mM methionine, 75 μM nitroblue tetrazolium chloride, 0.1 mM EDTA, and 50 mM sodium carbonate. Reactions were initiated by adding 60 μL of 100 μM riboflavin solution and placing the reaction mixtures under two 30 W fluorescent lamps for 15 min. The reaction was stopped by turning off the light and incubating the mixtures in darkness, and a non-irradiated reaction mixture served as the blank. Absorbance was recorded at 560 nm using a microplate reader (Multiskan SkyHigh Microplate Spectrophotometer, Thermo Fisher Scientific, Waltham, MA, USA).

Ascorbate peroxidase (APX, EC 1.11.1.11) was determined following the method of Erdogan et al. (2016) [47]. An aliquot (200 µL) of enzymatic extract was added to 3 mL of 50 mM phosphate buffer (PBS, pH 7.0), 0.5 mM ascorbic acid, and 0.1 mM H_2_O_2_. Absorbance at 290 nm was measured for 1 min using a microplate reader (Multiskan SkyHigh Microplate Spectrophotometer, Thermo Fisher Scientific, Waltham, MA, USA).

Catalase (CAT, 1.11.1.6) activity was determined by monitoring the initial rate of H_2_O_2_ disappearance. The reaction mixture for CAT assay (3 mL) contained 10 mM potassium phosphate buffer (pH 7.0), 100 µL of enzymatic extract, and 0.035 mL of 3% H_2_O_2_. The decrease in H_2_O_2_ was followed as reduction in absorbance at 240 nm, and activity was calculated using the extinction coefficient (40 mM^−1^ cm^−1^) for H_2_O_2_, according to Velikova et al. (2000) [48].

Peroxidase (POD, EC 1.11.1) activity was assessed by monitoring the rise in absorbance at 470 nm in a reaction mixture containing 50 mM phosphate buffer (pH 5.5), 1 mM guaiacol, and 0.5 mM H_2_O_2_. One unit of POD activity was defined as the enzyme amount causing an absorbance increase of 0.01 min^−1^ based on Erdogan et al. (2016) [47].

#### Non-Enzymatic Antioxidant Activity Determination

Total phenolic compounds and total flavonoids were determined using an aluminum chloride colorimetric assay as described by Yáñez et al. (2025) [49]. Quantification was performed using calibration curves constructed with gallic acid (for total phenols) and quercetin as a standard for estimating total flavonoid content. Results were expressed as g of gallic acid equivalents per 100 g fresh weight (GAE 100 g^−1^ FW) and g quercetin equivalents per 100 g fresh weight (QE 100 g^−1^ FW), respectively.

### 4.7. Statistical Analysis

The effects of endophyte inoculation and temperature, and their interaction, on each response variable were analyzed using two-way analysis of variance (ANOVA). Response variables included plant performance variables (total, shoot, and root biomass, and shoot:root ratio), oxidative stress markers (H_2_O_2_ and malondialdehyde, MDA), enzymatic antioxidant activities (superoxide dismutase, SOD; catalase, CAT; ascorbate peroxidase, APX; and peroxidase, POD), and non-enzymatic antioxidants (flavonoids and total phenols). Prior to analysis, data were checked for normality and homogeneity of variances. When necessary, data were transformed to meet ANOVA assumptions. Multiple comparisons among means were performed using Tukey’s honestly significant difference (HSD) test at *p* < 0.05. All analyses were conducted using GraphPad Prism 10 (GraphPad Software Inc., San Diego, CA, USA), and figures were generated using the same software.

## 5. Conclusions

Symbiosis with endophytic fungi isolated from desert environments increased heat stress tolerance in tomato plants. This effect was evident through greater shoot and root biomass, together with lower MDA and H_2_O_2_ levels. This response was associated with increased activity of SOD, CAT, and APX (while POD responded primarily to temperature), as well as higher levels of non-enzymatic antioxidants (phenols and flavonoids), indicating an endophyte-mediated modulation of antioxidant responses under heat stress. However, differences among fungal inoculation treatments indicate that plant–endophyte associations vary depending on the fungal partner. Further studies integrating physiological, molecular, and metabolomic approaches are required to better characterize these associations. A deeper understanding of plant-endophyte associations may contribute to improving crop tolerance to heat stress under climate change scenarios.

## Data Availability

The raw data supporting the conclusions of this article will be made available by the authors upon request.

## Supplementary Materials

The following supporting information can be downloaded at: https://www.mdpi.com/article/10.3390/plants15152311/s1, Figure S1: Assessment of symbiosis establishment. Verification of endophytic fungal colonization in tomato (*Solanum lycopersicum* L.) roots. Bright-field microscopy images: (A) non-inoculated control roots, (B) roots inoculated with *Talaromyces minioluteus* (E1+), and (C) roots inoculated with *Serendipita indica* (E2+), stained with lactophenol blue. Confocal microscopy images: (D) non-inoculated control roots, (E) roots inoculated with *T. minioluteus* (E1+), and (F) roots inoculated with *S. indica* (E2+), stained with WGA-AF^TM^ 488. Fungal structures (e.g., intra- and intercellular hyphae) are visible only in inoculated treatments, confirming successful colonization.
