# Supplementary material for: Desert Endophytic Fungi Differentially Modulate Reactive Oxygen Species and Antioxidant Responses to Heat Stress in Tomato
Source: Plants (Basel). 2026 Jul 28;15(15):2311. doi: 10.3390/plants15152311 (PMC13468373; doi:10.3390/plants15152311)
Supplement: Supplementary file 1 [file plants-15-02311-s001.zip › plants-4418477-supplementary.pdf]

Supplementary Materials

Assessment of symbiosis establishment

Root fungal colonization was assessed by combining fungal re-isolation, identification of recovered isolates, and microscopic visualization of root tissues. Roots were collected from three biological replicates per treatment, cut into 2–3 cm fragments, and surface-disinfected by sequential immersion in 70% ethanol for 30 s, 1% sodium hypochlorite for 3 min, and 95% ethanol for 5 min, followed by three rinses with sterile distilled water. Fragments were dried on sterile filter paper, cut into 1–1.5 cm sections, and plated on PDA medium supplemented with 100 µg mL^{-1} streptomycin sulfate (Schulz et al., 1993). Plates were incubated at 28 ºC until fungal mycelium emerged from root tissues, and emerging mycelia were then subcultured onto fresh PDA plates for purification and subsequent identification by molecular verification of fungal DNA amplification with ITS markers (Morales-Quintana et al. 2022).

Identification of fungal isolates from roots was performed through a combination of morphological and molecular analyses. Morphological characterization included examination of reproductive structures (conidiophores and conidia) based on taxonomic keys (Arx, 1981; Barnett & Hunter, 1998; Leslie & Summerell, 2005). Microscopic observations were performed using lactophenol blue (Morales-Quintana et al. 2022). For this, 30 root fragments (10–15 mm) were cleared in 5% KOH at 90 °C for 15 min, acidified in 1% HCl at 20 °C overnight, and then stained with 0.05% (w/v) lactophenol blue in lactoglycerol (lactic acid:glycerol:water, 1:1:1), at 90 °C for 30 min and observed under a light microscope (iScope, Euromex, Netherlands).

To further confirm root colonization, fungal structures within root tissues were visualized using staining techniques. Root fragments from three plants per treatment were subjected to staining with trypan blue (Koske & Gemma, 1989) and WGA-AF™488 (Gaber et al., 2023). Roots were subsequently cleared in 50% glycerol for 24 h and stored in lactic acid. For WGA-AF™488 staining, root fragments (5–10 mm) were incubated with 2 µg mL^{-1} WGA-AF™488 for 10 min, and fungal structures were visualized using Carl Zeiss LSM 700 confocal microscope. Microscopic observations confirmed successful colonization by *T. minioluteus* (E1+) and *S. indica* (E2+) in all inoculated plants, evidenced by the presence of characteristic endophytic structures, including

hyphae within root tissues. Roots of inoculated plants harbored intercellular hyphae of both *fungal species* (Figure S1).

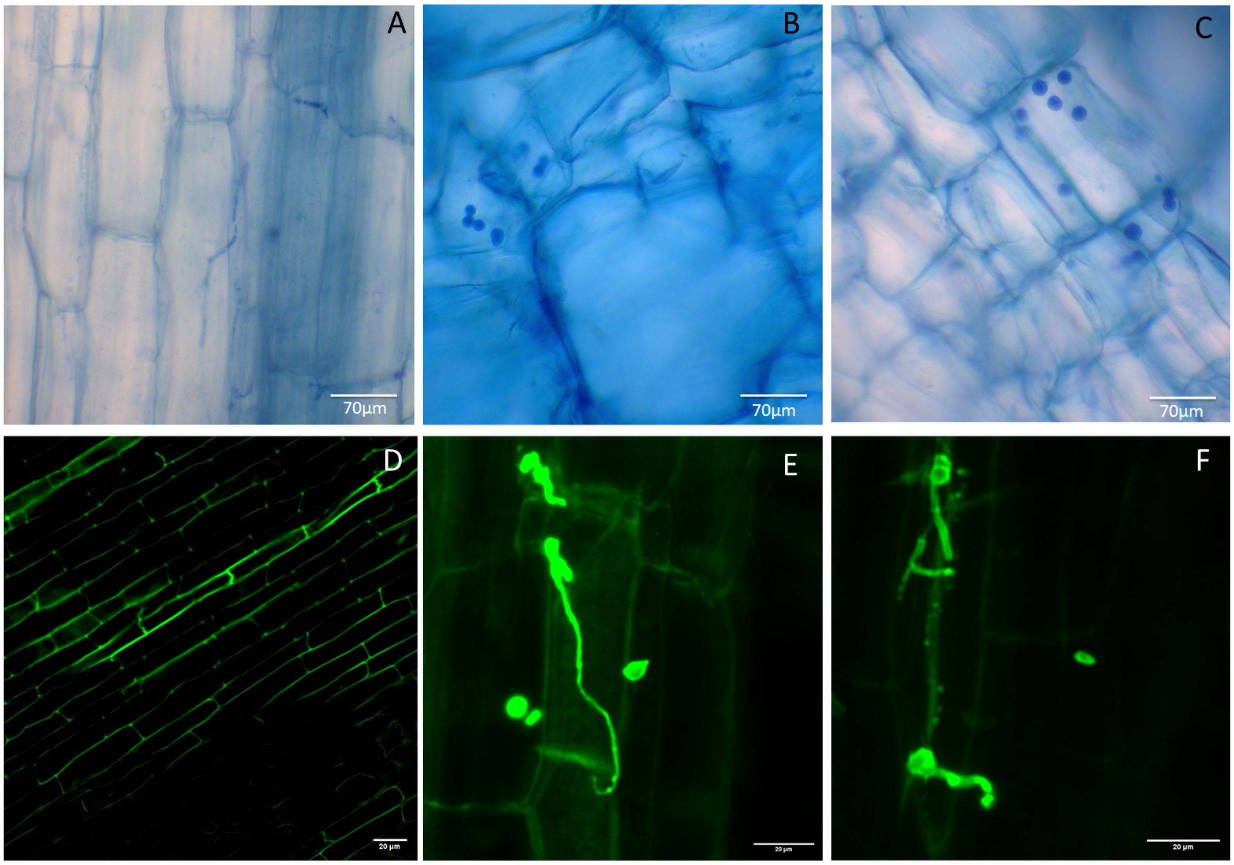

Figure S1. Verification of endophytic fungal colonization in tomato (*Solanum lycopersicum* L.) roots. Bright-field microscopy images: (A) non-inoculated control roots, (B) roots inoculated with *Talaromyces minioluteus* (E1+), and (C) roots inoculated with *Serendipita indica* (E2+), stained with lactophenol blue. Confocal microscopy images: (D) non-inoculated control roots, (E) roots inoculated with *T. minioluteus* (E1+), and (F) roots inoculated with *S. indica* (E2+), stained with WGA-AF™ 488. Fungal structures (e.g., intra- and intercellular hyphae) are visible only in inoculated treatments, confirming successful colonization.
